# Lack of direct involvement of a diazepam long-term treatment in the occurrence of irreversible cognitive impairment: a pre-clinical approach

**DOI:** 10.1038/s41398-021-01718-8

**Published:** 2021-12-03

**Authors:** Louise Carton, Candice Niot, Maéva Kyheng, Maud Petrault, Charlotte Laloux, Camille Potey, Marie Lenski, Régis Bordet, Julie Deguil

**Affiliations:** 1 Univ. Lille, Inserm, CHU Lille, Lille Neuroscience and Cognition, Degenerative and Vascular Cognitive Disorders, UMR-S1172, 59000 Lille, France; 2 Pharmacy Service, Arras Hospital Center, 62000 Arras, France; 3 Univ. Lille, CHU Lille, ULR 2694 - METRICS: Évaluation des Technologies de Santé et des Pratiques Médicales, 59000 Lille, France; 4 Département de Biostatistiques, CHU Lille, 59000 Lille, France; 5 Univ. Lille, CNRS, Inserm, CHU Lille, Institut Pasteur de Lille, US 41 - UMS 2014 - PLBS, Lille In vivo Imaging and Functional Exploration, 59000 Lille, France; 6 Univ. Lille, CHU Lille, Institut Pasteur de Lille, ULR 4483 - IMPECS – Impact de l’Environnement Chimique sur la Santé, 59000 Lille, France

**Keywords:** Pharmacodynamics, Diseases, Pathogenesis, Learning and memory

## Abstract

Several observational studies have found a link between the long-term use of benzodiazepines and dementia, which remains controversial. Our study was designed to assess (i) whether the long-term use of benzodiazepines, at two different doses, has an irreversible effect on cognition, (ii) and whether there is an age-dependent effect. One hundred and five C57Bl/6 male mice were randomly assigned to the 15 mg/kg/day, the 30 mg/kg/day diazepam-supplemented pellets, or the control group. Each group comprised mice aged 6 or 12 months at the beginning of the experiments and treated for 16 weeks. Two sessions of behavioral assessment were conducted: after 8 weeks of treatment and after treatment completion following a 1-week wash-out period. The mid-treatment test battery included the elevated plus maze test, the Y maze spontaneous alternation test, and the open field test. The post-treatment battery was upgraded with three additional tests: the novel object recognition task, the Barnes maze test, and the touchscreen-based paired-associated learning task. At mid-treatment, working memory was impaired in the 15 mg/kg diazepam group compared to the control group (*p* = 0.005). No age effect was evidenced. The post-treatment assessment of cognitive functions (working memory, visual recognition memory, spatial reference learning and memory, and visuospatial memory) did not significantly differ between groups. Despite a cognitive impact during treatment, the lack of cognitive impairment after long-term treatment discontinuation suggests that benzodiazepines alone do not cause irreversible deleterious effects on cognitive functions and supports the interest of discontinuation in chronically treated patients.

## Introduction

Benzodiazepines, allosteric agonists of the γ-aminobutyric acid (GABA) A receptor, are a class of psychotropic drugs commonly prescribed to treat acute anxiety and/or insomnia [1]. The worldwide global prevalence use of benzodiazepines ranges from 2.6 to 12.5% [2] and increases with age [3, 4], with a mean or median age of use around 55 years [5–7]. Adverse effects like cognitive deterioration, paradoxical response, risk of falling, misuse, and dependence have led several countries to reduce prescription duration [2]. However, despite general warnings from drug regulatory agencies, the long-term use and overprescription of benzodiazepines persist, particularly in the aging population. The major concern arising from observational studies in this population is an increased risk of irreversible cognitive deficits leading to dementia after long-term use of benzodiazepines [8–10]. According to these studies, the risk magnitude may be influenced by treatment duration, dosage used, and probably benzodiazepine half-life [11]. However, the link between the long-term use of benzodiazepines and neurocognitive disorders remains controversial [12–14]. Two hypotheses can be formulated to explain this discrepancy. First hypothesis: benzodiazepines have no influence on age-related cognitive decline and these conflicting results can simply be explained by methodological issues such as the protopathic bias, since the main indications for benzodiazepines (anxiety, insomnia) can also be prodromes of dementia [12, 13].

Second hypothesis: benzodiazepines have a negative impact on brain aging processes leading to the emergence of reversible or irreversible brain changes. In the context of pathological aging, i.e., in patients with an underlying neurodegenerative disease, benzodiazepines may decrease the cognitive reserve and accelerate the onset and presentation of the disease. In the context of normal aging, i.e. in patients without any underlying disease, benzodiazepines could prime irreversible cognitive impairment and contribute to dementia through several possible mechanisms: (i) GABA/glutamate imbalance, (ii) impact on neural plasticity and survival, (iii) hypoxic mechanisms through respiratory depression [15–17]. It is this latter assumption that we propose to explore in this preclinical work.

Indeed, the direct long-term effects of benzodiazepines on cognition cannot be clearly demonstrated by epidemiological studies where the temporality criterion remains uncertain. Furthermore, prospective clinical trials are not feasible due to evident ethical issues. In this context, animal models represent a relevant approach to overcome clinical limitations by a tight control of the confounding factors that may skew clinical outcomes.

The available studies led on rodents demonstrated psychopharmacological responses similar to humans after a single or repeated benzodiazepine administrations in terms of therapeutic properties (i.e. sedative, anxiolytic, anticonvulsant and muscle relaxant effects) and adverse effects in particular amnesic effects [18, 19]. Overall, long-term explicit memory was shown to be particularly affected, which mainly results from an alteration in the acquisition stage (encoding phase) of the memory process [20]. As observed in humans, animal studies evidenced the contradictory effects of benzodiazepines on memory retrieval processes [20]. Furthermore, data about the effects of long-term benzodiazepines use on cognition in pre-clinical models are missing. Most of these deficits have been characterized after a single administration and may vary according to dose [21, 22].

This study was designed to evaluate whether the use of diazepam over a period of time considered inappropriate with regard to the recommendations of French good clinical practices (set at 12 weeks) [23], may interact with brain aging processes and induce irreversible effects on cognitive functions, i.e. persisting after treatment withdrawal. To improve the translational value of this preclinical study, we have considered several factors pointed out by epidemiological studies as likely to increase the risk of long-term cognitive effects such as a prolonged duration of treatment and use of a long-acting benzodiazepine [11, 24]. We also studied the influence of two other factors: (i) the dose of drug and (ii) the age at the time of drug exposure. The choice of study design, doses and age range was guided by the available clinical and preclinical data, with the aim of studying whether the dose of benzodiazepine used long-term during periods of life variably affected by brain aging processes impacts the evolution of cognitive capacities.

## Materials and methods

### Subjects and handling

One hundred and five C57BL/6JRj male mice from Janvier Laboratories (Le Genest St Isle, France) aged 6 (n = 58) and 12 months (*n* = 47), called mature and middle-aged mice, were used in this study. These ages were chosen to reflect the impact of treatment not only in adulthood (7–11 months) but also in aging adults (13–17 months) during the age period in which the early manifestations of cerebral aging set in, as described by Singhal et al. [25].

The mice were housed in groups of 4–5 per cage in an enriched environment and were maintained under conditions of constant temperature (19–24 °C) and humidity (60–70%) under a 12-h light/dark cycle. Animals were allowed to acclimate to the laboratory for 10 days before any experimental manipulation. All experiments were approved by the national Ethical Committee in Animal Experimentation (Comité d’éthique en Expérimentation Animale Nord-Pas de Calais CEEA no.75) and by the French Ministry for Education and Research (agreement number: 2018060818218219 v4) and were performed in strict compliance with the European Union Directive 2010/63/EU. Experiments were reported in accordance with the ARRIVE guidelines for reporting experiments involving animals.

### Treatment administration

Diazepam was incorporated into the diet to avoid stress and damage from chronic force feeding. Mature (6 months of age) and middle-aged (12 months of age) mice were randomly assigned to one of the three different groups constituted as follows:

- A control group with normal diet (CTL) of 25 mature and 20 middle-aged mice;
- A diazepam-supplemented diet group at 15 mg/kg/day (D15) of 15 mature and 14 middle-aged mice;
- A diazepam-supplemented diet group at 30 mg/kg/day (D30) of 18 mature and 13 middle-aged mice.

The daily dose of 15 mg/kg of diazepam in mice was determined using the body surface area-based adjustment method as described by Nair and Jacob [26], corresponding approximately to the maximum recommended human dose of 1 mg/kg/day. The dose of 30 mg/kg/day takes into account metabolic changes or tolerance phenomena that may occur during repeated drug administration. Throughout the 16 weeks of treatment, food consumption and body weight were measured to weekly adjust diazepam concentration in the food. Access to water was ad libitum for all groups. For the preparation of pellets, diazepam tablets (ARROW Laboratory) were crushed and mixed into the powdered diet (Scientific Animal Food and Engineering (SAFE), A04, France). The CTL group was fed with SAFE pellets. In all the diazepam-treated groups, the daily oral dose was gradually increased in increments of 7.5 mg/kg every 3 days up to the final dose and gradually reduced at the end of the 16-week period, to avoid any withdrawal syndrome.

### Experimental design

**Fig. 1:**
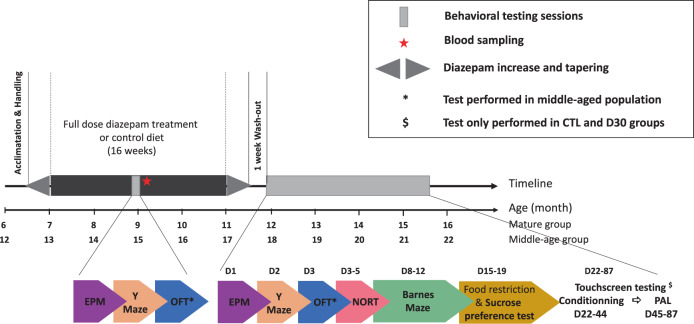
Study design, timeline, and methods of the study. The top line shows the timing of treatment and behavioral sessions. The bottom line shows the age of mice (mature and middle-aged) at the different stages of the experiment. The lower part of the figure indicates the behavioral tests performed for each assessment session and their order. EPM elevated plus maze, NORT novel object recognition test, OFT open field test, PAL paired-associate learning.

Two sessions of behavioral assessment were carried out using a battery of tests performed in the order shown in Fig. 1. The first one was performed after 8 weeks of treatment (mid-treatment) to assess in our experimental conditions the amnesic effect described in humans, using the Y maze spontaneous alternation test. The elevated plus maze (EPM) test was also performed to control the anxiety level of mice. The open field test (OFT) was secondarily integrated to evaluate the sedative effect of diazepam in the middle-aged mice, an age group subject to a high rate of exclusion in the memory task. The second behavioral assessment was performed after 16 weeks of treatment, after dose tapering, and a 1-week wash-out period to assess the long-term cognitive effects after diazepam withdrawal. At the beginning of this second assessment phase, the mice were 11.5 months and 17.5 months old. This post-treatment test battery was enriched with the novel object recognition (NOR) test, the Barnes maze test, and the touchscreen paired-associate learning (PAL) task. Given the time constraints inherent to this latter test, we subjected only the CTL and D30 groups of mice to the PAL task.

For most of the tests, assessments were conducted between 8:30 a.m. and 1:00 p.m. during the light period of the light/dark cycle. However, to homogenize the influence of the circadian cycle on the performances of mice in the different groups, we systematically modified the running order of animals for each test session. For the tests performed throughout the day (mainly the Barnes maze test and the touchscreen test), we also alternated the order of the animals during the training days.

The investigator was not blinded to the group allocation during the experiment, but the outcomes were analyzed by investigators unaware of the experimental conditions.

The sequence of tests and test durations are shown in Fig. 1.

### Behavioral testing

#### EPM test

The EPM test was used to measure the anxiety level of mice. Mice were placed in the middle of the maze consisting of two open (stressful) and two enclosed (protecting) arms (36.5 cm × 6 cm) forming an elevated cross (positioned 50 cm above the floor) and allowed to explore the maze for 10 min. The time spent in each arm was recorded with a video tracking system (Ethovision XT 14.0, Noldus, Wageningen, The Netherlands) to calculate the percentage of time spent in the open arms, used as an index of anxiety behavior.

#### Open field test

The OFT was used to assess spontaneous locomotor activity with an open-field infrared actimeter (Bioseb, Vitrolles, FR). Mice were placed in the center of the apparatus (45 × 45 × 34 cm^3^) and allowed to explore freely for 10 min. The main outcome was the total distance traveled (in cm) recorded by two rows of infrared photocells and processed with the Actitrack software (Bioseb, Vitrolles, FR).

#### Y maze spontaneous alternation test

The Y maze spontaneous alternation test was used to assess working memory and performed as previously described [27]. The mice were allowed to freely explore the three arms of the maze during 8 min. The sequence of arm entries was manually noted to calculate the number of alternations defined as a sequence of three consecutive visits to different arms. The main outcome was the percentage of spontaneous alternations calculated using the formula: number of alternations/(total number of arm visits − 2) × 100. We excluded from the analysis animals that did not perform a minimum of 16 entries during the 8-min task.

#### NOR test

The NOR test was used to assess visual recognition memory and performed as previously described [28]. The test took place over 3 days with a phase of arena habituation (without and with objects) for the first 2 days, followed by the test day including acquisition and retention trials. During the acquisition trial, mice were free to explore two identical objects (called familiar objects) for 15 min. After a 1-h break (during which mice were returned to their cages), a 5-min retention trial was performed in which one of the two familiar objects was replaced by a novel one. The main outcome was the discrimination index (DI), calculated as follows: DI = (TN − TF)/(TN + TF), where TN is the exploration time of novel object, and TF the exploration time of familiar object. Animals spending <20 s exploring objects during the acquisition trial were excluded from the analysis. This test was video-tracked and analyzed with Ethovision XT 14 software (NOLDUS, Wageningen, NL).

#### Barnes maze test

The Barnes maze test was used to explore spatial reference learning and memory as previously described [29]. In a white elevated circular platform with 40 holes, an escape box was placed under a target hole (always constant for a given mouse) randomly allocated among mice. During a habituation phase, the animal was guided toward the escape box, where it stayed for 2 min. During the learning phase, mice performed 4 trials of 3 min each per day, with a 15-min break between trials, for 4 consecutive days, in order to learn the location of the escape box using visual extra-maze cues. On the fifth day, a probe trial of 90 s, in which the escape box was removed, was conducted to assess spatial reference memory performances calculated as the percentage of time spent in the target quadrant (i.e., the quadrant centered on the target hole). To assess spatial reference learning we used the average daily total escape latency during learning trials. The EthoVision XT 14 software (Noldus, The Netherlands) was used for data analysis.

#### Touchscreen different PAL (dPAL) task

Visuospatial learning was evaluated in the dPAL task as previously described [29]. Using Touchscreen operant chambers and ABET software (Campden Instruments Ltd, UK), mice were tested for their ability to associate different images with specific spatial locations on the tactile screen. Prior to touchscreen testing, appetitive behavior toward sweet fluids was assessed in a sucrose preference test. Mice were first trained to collect free drops of chocolate flavored milk that were then associated with correct nose-poke responses on the screen during the stimuli presentation as a reward. For the dPAL task, one of the six possible trial types was randomly presented on the screen (i.e., six possible pairs of images among three different images in three distinct locations on the screen). For each trial type, only one visual stimulus was presented in its correct location (+), a second visual stimulus was presented in one of the two incorrect locations (−), and the third location remained blank. The main outcome was the visuospatial memory, measured as the mean percentage of correct responses (nose-poke on the correct stimulus) per session (average of 3 consecutive trials) over 30 days.

### Dosages

Blood samples were collected from the retro-orbital sinus at mid-treatment, after completion of the behavioral assessment (approximately 9 weeks after starting the full dose), to measure the plasma concentrations of diazepam and its metabolites (nordiazepam, oxazepam, temazepam), using a validated liquid chromatography tandem mass spectrometry (LC-MS/MS) analysis method. The limit of quantification of diazepam and its metabolites is 50 ng/mL. For protein precipitation, plasma (50 μL) was mixed and centrifuged (14,000 rpm, 4 °C, 10 min) after addition of 200 μL of a solution of acetonitrile containing methylclonazepam (Alsachim, France) at 5 mg/L, as an internal standard. In all, 50 μL of the supernatant was added to 200 µL of high aqueous solvent, and 15 µL of this mixture were injected. Chromatography was performed on an H-Class Acquity UPLC system (Waters, Milford, MA) equipped with an Acquity HSS C18 column (1.8 µm, 150 × 2.1 mm, Waters, Milford, MA). The column temperature and flow rate were set at 50 °C and 0.4 mL/min during all of the solvent gradient. The UPLC system was coupled to a TQ Detector (Waters, Milford, MA) through an electrospray interface. The instrument was operated in multiple reaction monitoring mode. Data processing was performed through the MassLynx software (version 4.2, Waters).

### Statistical analysis

We grouped for the analysis and figures all animal receiving a same dose of treatment. Categorical variables were expressed as frequencies and percentages. Quantitative variables were expressed as mean (standard deviation) or median (interquartile range) for non-normal distributions. The normality of distributions was assessed graphically and using Shapiro–Wilk test. We compared mid-term quantitative variables between the three experimental groups using one-way analysis of variance (ANOVA); post hoc pairwise comparisons were done using linear contrast after Bonferroni correction. Variables with a non-normal distribution were log-transformed. The rate of exclusion was compared between treatment groups using Fisher tests in overall and according to the age of mice. In addition, the relationship between mid-term quantitative parameters was appreciated using Spearman’s correlation coefficients.

The same analyses (ANOVA and post hoc tests, Fisher tests) were done to compare long-term quantitative variables between groups. We further compared the intra-mice variability in spatial reference learning and memory assessment between the three groups by using a linear mixed model, including time, groups, and time × groups interaction as fixed effects. In case of a significant interaction between time and groups, a post hoc comparison between each group was done using linear contrast after Bonferroni correction. Finally, we investigated the heterogeneity of the treatment effect size for outcomes according to age by introducing a multiplicative term into regression models (ANOVA and linear mixed model). Statistical testing was done at the two-tailed *α* level of 0.05. Data were analyzed using the SAS software package, release 9.4 (SAS Institute, Cary, NC).

## Results

### Plasma concentrations of diazepam and its metabolites

The plasma profiles showed that diazepam was rapidly metabolized (only trace amounts were found in some groups) to its active metabolites, including nordiazepam and oxazepam (Table 1). The assay showed higher plasma levels of oxazepam than nordiazepam regardless of age or dose. In addition, the accumulation of oxazepam was significantly greater in the middle-aged group compared to the mature group, *p* = 0.002 and also greater for both oxazepam and nordiazepam in the D30 group vs the D15 group, *p* < 0.001.

**Table 1:** Plasma concentration of diazepam and its metabolites (ng/mL) measured at mid-treatment time point in mature and middle-aged mice chronically treated with 15 mg/kg/day (D15) or 30 mg/kg/day (D30) of diazepam.

		Diazepam (ng/mL)	Nordiazepam (ng/mL)	Oxazepam (ng/mL)
D15	Mature	0	33 (27; 148)	432 (359; 498)
	Middle-aged	5.9 (5.4; 9.7)	348 (277.3; 469)	1233^a^ (919.8; 1465)
D30	Mature	0	79.8^b^ (57.9; 127.3)	755.8^b^ (621.5; 880.8)
	Middle-aged	0 (0; 11.8)	259^b^ (178.5; 396)	2262^a,b^ (1754; 2469)

Values are median ± interquartile range (IQR).

^a^Oxazepam level in middle-aged vs mature group, *p* = 0.002.

^b^Nordiazepam and oxazepam levels in D30 vs D15 group, *p* < 0.001.

### Mid-treatment consequences of a chronic diazepam administration

#### Anxiety assessment in the EPM test

**Fig. 2:**
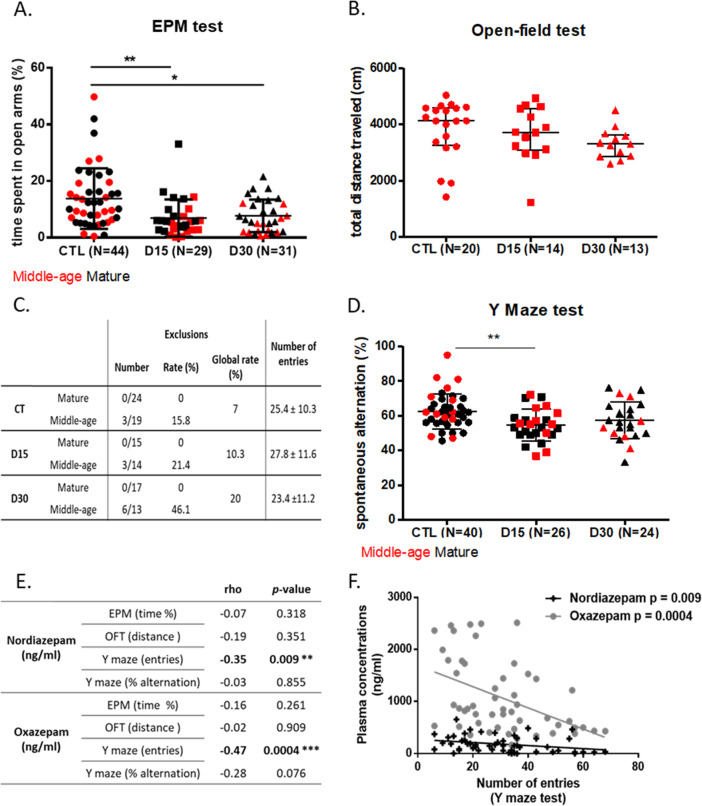
Mid-treatment consequences of a chronic diazepam administration on: anxiety, spontaneous locomoto ractivity and working memory. The effects of 2 months of a 15 mg/kg/day (D15), a 30 mg/kg/day (D30) of diazepam treatment, or control conditions (CTL) on: **A** the percentage of time spent in open arms of mature and middle-aged mice during the elevated plus maze (EPM) test, **B** the total distance traveled during the open field test (OFT) performed on middle-aged mice, **C** the number of entries and the exclusion rate of mature and middle-aged mice in the Y maze test, **D** the percentage of spontaneous alternations of mature and middle-aged mice during the Y maze test, **E** table for Spearman correlation coefficients, and **F** relationship between the number of entries in the Y maze spontaneous alternation task and plasma nordiazepam or oxazepam concentrations. Mature mice are shown in black and middle-aged mice in red. Results are expressed as mean +/− SD for the Y maze test and as median (IQR) for the EPM and OFT, *p < 0.05, **p < 0.01, ***p < 0.001. The statistical analysis was performed using one-way ANOVA followed with Bonferroni’s post hoc comparison and Spearman test for the correlation analysis.

The percentage of time spent in the open arms of the EPM test was significantly smaller in the D15 and D30 groups in comparison to the CTL group, respectively, 5.6% (2.7; 9.9); 6.3% (2.1; 12.1); 11.3% (5.8; 17.7), *p* = 0.001, suggesting a greater level of anxiety in treated mice (Fig. 2A). The age did not influence the impact of treatment on the performances in the EPM test (*p* value of interaction = 0.14).

#### Spontaneous locomotor activity assessment in the OFT

The total distance traveled by middle-aged mice in this test were not significantly changed by the diazepam treatment, respectively, 4125 cm (3291; 4572), 3715 cm (3119; 4552), and 3299 cm (2876; 3588) for the CTL, D15, and D30 groups (*p* = 0.64) (Fig. 2B).

#### Working memory assessment in the Y maze spontaneous alternation test

As indicated in the table (Fig. 2C), respectively, 3/43 (7.0%), 3/29 (10.3%), and 6/30 (20%) animals in the CTL, D15, and D30 groups were excluded because they did not reach the required minimum number of total entries. The comparison of the exclusion rates did not show any significant difference between groups both in the whole group (*p* = 0.31) and in the middle-aged subgroup (*p* = 0.19) suggesting that treatment had no impact on the exclusion rate regardless of animal age. However, age seems to play a role on this parameter since, regardless of the group, all excluded animals belonged to the middle-aged subpopulation (Fig. 2C). The comparison of the total number of entries that reflects the exploratory capacity did not reveal any difference between groups (*p* = 0.38) (Fig. 2C).

The percentage of alternations was significantly decreased in the D15 group, but not in the D30 group, in comparison to CTL, respectively, 54.6 ± 9.2%, 57.3 ± 10.7%, and 63.1 ± 10.7%, for the D15, D30, and CTL groups (*p* = 0.005). Age did not influence the effect of treatment on test performance (*p* value of interaction = 0.19) (Fig. 2D).

#### Relationship between drugs plasma concentrations and mid-treatment behavioral performance

The correlation analysis showed no association between nordiazepam and oxazepam plasma concentrations and the level of anxiety defined by the percentage of time in open arms in the EPM test (*p* = 0.32 and *p* = 0.26, for nordiazepam and oxazepam, respectively) or with the exploratory activity defined by the total distance traveled in the OFT (*p* = 0.35 and *p* = 0.91, respectively) (Fig. 2E). Results revealed a significant negative association between nordiazepam and oxazepam plasma concentrations and the number of entries in the Y Maze test (*p* = 0.009 and *p* = 0.0004, respectively) (Fig. 2E, F). No association was found between nordiazepam and oxazepam plasma concentrations and the percentage of alternation in Y maze task (*p* = 0.85 and *p* = 0.08, respectively).

### Long-term behavioral consequences of a chronic diazepam exposure after treatment withdrawal

#### Anxiety assessment in the EPM test

**Fig. 3:**
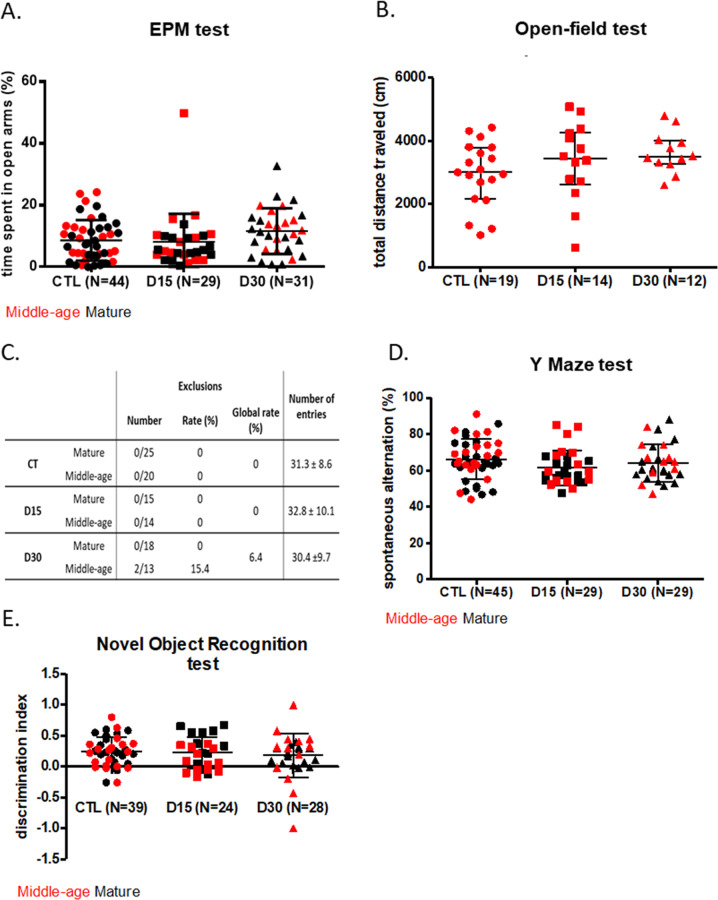
Long-term consequences of a chronic diazepam exposure after treatment withdrawal on: anxiety, spontaneous locomotor activity, working memory and visual recognition memory. The long-term effects of a chronic diazepam treatment after drug withdrawal on **A** the percentage of time spent in open arms of mature and middle-aged mice during the elevated plus maze (EPM) test, **B** the total traveled distance during the open field test (OFT) performed on middle-aged mice, **C** the number of entries and the exclusion rate of mature and middle-aged mice in the Y maze test, **D** the percentage of spontaneous alternation of mature and middle-aged mice during the Y maze test, and **E** the discrimination index during the novel object recognition (NOR) test. Mature mice are shown in black and middle-aged mice in red. Results are expressed as mean +/− SD for the EPM, Y maze spontaneous alternation, and NOR tests and as median (IQR) for the OFT.

The percentage of time spent in the open arms of the EPM was not significantly different between groups, respectively, 8.6 ± 6.5%, 8.2 ± 9.1%, and 11.6 ± 7.4% for the CTL, D15, and D30 groups, *p* = 0.098 (Fig. 3A).

#### Spontaneous locomotor activity assessment in the OFT

The comparison of the total distance traveled measured in the OFT in middle-aged mice did not highlight any difference between groups, respectively, 2973 cm (2403; 3701), 3445 cm (2718; 4238), and 3465 cm (3324; 3942) for the CTL, D15, and D30 groups (*p* = 0.21) (Fig. 3B).

#### Working memory assessment in the spontaneous alternation test

Only two middle-aged mice in the D30 group (2/31, 6.4%) were excluded because they did not reach the minimum number of total entries (Fig. 3C). The comparison of the exclusion rates in the different experimental groups did not show any difference suggesting that the treatment had no impact on this exclusion rate. In addition, the comparison of the total number of entries also did not evidence any significant difference between groups (*p* = 0.60) (Fig. 3C).

No difference was seen between groups in the spontaneous alternation rate, respectively 66.1 ± 11.0%, 61.5 ± 9.6%, and 64.0 ± 10.2% for the CTL, D15, and D30 groups (*p* = 0.19) (Fig. 3D). The age of mice did not modify the effect of treatment on working memory (*p* value of interaction = 0.74).

#### Visual recognition memory assessment in the NOR test

Respectively, 6/45 (13.3%), 5/29 (17.2%), and 3/31 (9.7%) of animals across the CTL, D15, and D30 groups were excluded because they did not sufficiently explore the objects during the acquisition phase. The comparison of the exclusion rates did not show any significant difference between the groups (*p* = 0.81).

No differences were seen between groups on the discrimination index, respectively 0.24 ± 0.24, 0.22 ± 0.26, and 0.18 ± 0.35 for the CTL, D15, and D30 groups (*p* = 0.65), without any age effect (*p* value of interaction = 0.16) (Fig. 3E).

#### Spatial reference learning and memory assessment in the Barnes maze test

**Fig. 4:**
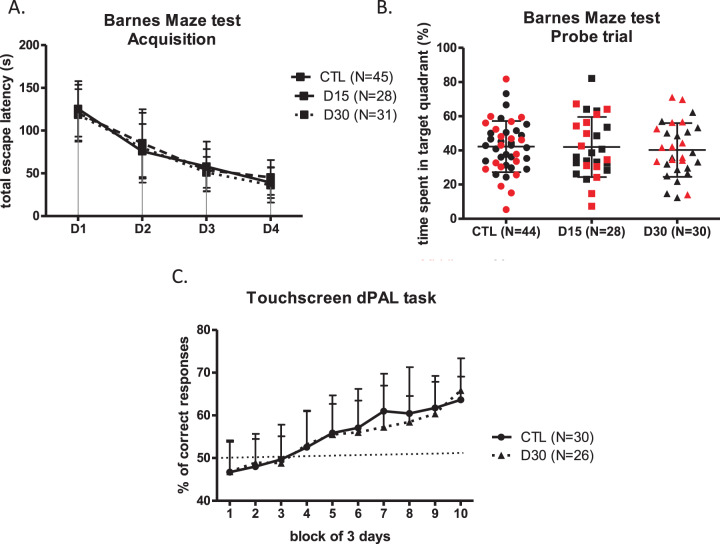
Long-term consequences of a chronic diazepam exposure after treatment withdrawal on: spatial reference learning and memory and visuospatial learning. The long-term effects of a chronic diazepam treatment after drug withdrawal on **A** the mean total escape latency during the learning session and **B** the time spent in target quadrant during the probe trial of the Barnes Maze test and **C** percentage of correct response performed by mice in the dPAL task. Mature mice are shown in black and middle-aged mice in red. Results are expressed as mean +/− SD.

No significant effects were observed when comparing the learning curves of the D15 and D30 groups with the CTL group for total escape latency (*p* = 0.70) (Fig. 4A). The comparison of the percentage of time spent in the target quadrant did not show any differences between the treated and non-treated groups, respectively 42.2 ± 15.0%, 41.9 ± 17.6%, and 40.3 ± 15.7% for the CTL, D15, and D30 groups (*p* = 0.86) (Fig. 4B).

#### Visuospatial learning assessment in the dPAL task

Only 6 mice (6/13; 46.1%) in the middle-age CTL group and 8 (8/13; 61.5%) in the middle-age D30 group completed successfully the operant conditioning phase and therefore were evaluated in the dPAL task.

In contrast, both mature CTL and D30 mice succeeded in the operant conditioning phase. The learning process was supported by the percentage of correct responses increasing along with test duration for both groups (*p* < 0.0001). No intergroup difference was evidenced during the whole assessment period (Fig. 4C).

## Discussion

In this study, we explored whether a long-term administration of diazepam may lead to lasting changes in cognitive outcomes after treatment discontinuation, focusing on the influence of dose- and age-related factors in a mouse model. In line with our hypothesis that diazepam may interact with brain aging processes to induce irreversible cognitive impairment, we chose two age groups that would be differently affected by cognitive aging processes at the time of diazepam treatment (i.e., 7–11 and 13–17 months) [30]. The design of the study, the doses of diazepam used (15 and 30 mg/kg/day) and the 16-week treatment duration (4 weeks longer than the maximum recommended duration in France) were also guided by evidence from clinical practice.

Our results confirmed the amnesic effect of diazepam on memory performances in the group of mice receiving 15 mg/kg/day of diazepam but we did not demonstrate any long-lasting impairment after treatment withdrawal in a range of cognitive domains, including working memory, spatial reference learning and memory, visual recognition memory, and visuospatial learning.

Diazepam is a long-acting benzodiazepine metabolized in both humans and rodents by CYP450 enzymes into the two active metabolites nordiazepam and temazepam, both further metabolized into oxazepam [31–33]. In our experimental conditions, the plasma assay carried out at mid-treatment shows an extensive dose-dependent plasma accumulation of oxazepam, and to a lesser extent of nordiazepam, while diazepam was almost undetectable. This accumulation of metabolites was increased in the middle-age subpopulation compared to the mature group of mice. One could hypothesize that an age-dependent progressive impairment of liver and kidney functions underlies these differences, as these alterations have been described in aging rodents [34–36].

The discrepancy with the plasma profiles derived from human data (longer plasma half-life of diazepam) did not constitute an obstacle since the metabolites of diazepam display the same spectrum of pharmacological activity as diazepam and may induce similar memory deficits [37, 38]. Furthermore, mean plasma concentrations of nordiazepam and oxazepam were within the human therapeutic reference range [39] validating two crucial points of this study, i.e., the route of administration and the range of doses used.

Surprisingly, the measurement of anxiety levels performed a few days before mid-treatment blood sampling evidenced an increase in anxiety in groups treated with diazepam 15 and 30 mg/kg/day, shown by a lower exploration of the open arms. The anxiety levels were not correlated with nordiazepam or oxazepam plasma concentrations suggesting that this effect was not dose-dependent. The confounding influence of sedation on this result was ruled out by comparing the distances traveled by the mice during the task, which was not significantly different in the treated groups compared to the control group (data not shown). This outcome was further supported by results in the OFT performed in middle-aged mice, which, although showing a trend toward a dose-dependent decrease in the exploratory activity in the diazepam-treated animals, did not reveal any significant difference between groups. This anxiogenic-like effect may be related to a paradoxical effect of benzodiazepines as previously described for several positive GABA-A modulators, such as allopregnanolone, barbiturates, ethanol, and benzodiazepines [40, 41] including diazepam [42].

Regarding cognitive abilities, we demonstrated that an 8-week long treatment with diazepam 15 mg/kg/day induced working memory impairment in mice. These results are consistent with previous preclinical studies [43–46]. Spatial working memory performances rely on a functional anatomic network involving hippocampus–prefrontal cortex interactions. These two brain structures were shown to display a high density of benzodiazepine receptor sites in rodents and humans [47] and may play a major role in diazepam-induced cognitive impairment. The lack of effect in the 30 mg/kg/day diazepam-treated mice may be related to the higher rate of exclusion in this group, halving the subpopulation of tested middle-aged mice thus limiting statistical power. These exclusions correspond to insufficient activity of animals in the maze, which could be attributed to the combined influence of age and treatment. The influence of age was supported by the fact that all excluded animals belonged to the older population. It is noteworthy that this age effect was no longer found in this task during the post-treatment session due to the re-test effect. In parallel, the negative association found between the plasma levels of the two main diazepam metabolites (i.e., nordiazepam and oxazepam) and the total number of entries in the maze suggested an effect of diazepam explaining the dose-dependent increase in the exclusion rate in the treated groups. Although it is difficult to establish the exact nature of the behavioral changes involved, the results in the OFT and EPM tests, as well as the known effects of diazepam on cognition, argue for the contribution of both cognitive and non-cognitive changes. Thus, certain behaviors such as goal-directed behavior (i.e., motivation) controlled by emotional and cognitive processes could be particularly involved.

In the post-treatment evaluation part, the measurement of anxiety levels was not different between group regimens indicating a lack of effect of diazepam after treatment cessation on anxiety behavior. One may question a retest effect in the EPM procedure as raised in other studies [48, 49]. However, such an effect is expected to lead to an increase in anxiety-like behavior [48], which is not observed in our conditions, probably due to the 2 months inter-test interval.

The working memory impairment found under diazepam treatment was not observed anymore after drug discontinuation, suggesting a reversibility of these memory deficits. To further explore the long-term consequences of a diazepam treatment on a wider range of cognitive functions, we submitted animals to additional tasks assessing spatial reference learning and memory, visual recognition memory, and visuospatial learning. These tasks functionally engage multiple cerebral structures such as the hippocampus, the prefrontal cortex and the perirhinal cortex, likely to be the sites of the amnesic action of benzodiazepines in mice and humans [20, 50, 51]. To increase the translational value of our study, we combined the use of ethological tests commonly used in mouse cognitive phenotyping (i.e., Barnes maze test, NOR test) with a translational one, procedurally inspired from the human neuropsychological tests automated battery (CANTAB) through a touchscreen-based automated system. In addition to providing a good cross-species cognitive assessment tool, the dPAL task is a very sensitive tool for the early detection of memory impairment in humans [52]. The ethological tests did not allow us to show any impairment in spatial reference learning and memory or visual recognition memory after diazepam treatment withdrawal in both age groups (12–13- and 18–19-month-old mice) when compared with the control group. Although we did not assess the integrity of these memory functions during drug exposure to avoid repetition bias, previous preclinical findings have provided evidence for their alteration following diazepam administration [51, 53]. These results may imply that the long-term treatment with diazepam seems unlikely to durably compromise the functioning of brain structures involved in these tasks, including the hippocampus, the perirhinal cortex and the prefrontal cortex [54]. On the other hand, the outcomes of the Touchscreen test were fairly complex to interpret in the older mice population, since about half of these animals (in both the control and diazepam groups) did not achieve the set criterion allowing them to be tested in the dPAL task. While a lack of motivation for the sweet reward was ruled out by the sucrose preference test performed just before the beginning of the touchscreen paradigm (data not shown), this result underlined an age-related deficit in instrumental conditioning abilities consistent with previous clinical and preclinical reports [55] that did not appear to be influenced by diazepam exposure. Although it is more than likely that this finding prevented the detection of the effect of age × drug interaction on visuospatial learning abilities, the comparison of mice performances did not indicate a deleterious effect of high-dose diazepam exposure on this memory type.

## Conclusions

Taken together, our results provided evidence that diazepam alone was not able to durably impair memory skills and that both dose- and age-related factors did not influence the long-term cognitive outcomes. The exclusion of female mice from the study population is a pitfall in this work. Evaluating the effects of sex on the long-term cognitive consequences of diazepam would be an interesting continuation to this study. Indeed, a recent clinical study highlighted variations in both therapeutic response and adverse effects of diazepam depending on gender and hormonal state [56].

In line with certain recent epidemiological data, our study did not find a causal relationship between long-term diazepam use and the development of irreversible cognitive impairment [12–14]. Moreover, the analysis of age subpopulations did not show any influence of this factor on the cognitive outcome of treated animals, which seems to rule out the hypothesis that benzodiazepines have a detrimental influence on normal brain aging processes leading to accelerated cognitive decline.
